# Effect of Regulation and Education on Reptile-associated Salmonellosis

**DOI:** 10.3201/eid1103.040694

**Published:** 2005-03

**Authors:** Birgitta de Jong, Yvonne Andersson, Karl Ekdahl

**Affiliations:** * Swedish Institute for Infectious Disease Control, Stockholm, Sweden; † Karolinska Institutet, Stockholm, Sweden

**Keywords:** Salmonella, salmonella infections, salmonella serotypes, reptiles, turtles, European Union, zoonoses, research

## Abstract

Reptiles have become increasingly common as domestic pets, and with them reptile-associated *Salmonella* infections in humans. From 1990 to 2000, a total of 339 reptile-associated *Salmonella* cases were reported in Sweden. In 1996, as part of its efforts to adapt its import regulations to those of the European Union, Sweden no longer required certificates stating that imported animals were free of *Salmonella*. A subsequent increase was noted in the incidence of reptile-associated cases from 0.15/100,000 in the period 1990–1994 to 0.79/100,000 in 1996 and 1997. After a public education campaign directed toward the general public was begun through the news media, the incidence dropped to 0.46/100,000. Children were the most affected age group among patients (incidence 1.3/100,000). *Salmonella* *enterica* serotype Enteritidis was the most frequent serotype (24% of isolates), followed by *S.* Typhimurium (9% of isolates). Import restrictions and public information campaigns are effective public health measures against reptile-associated salmonellosis.

## Abstract 2

Import restrictions and public awareness campaigns are effective against this common childhood infection.

Salmonellosis is an important worldwide health problem, affecting both humans and animals. In the United States, *Salmonella* causes an estimated 1.4 million episodes of infection and 400 deaths annually in humans (*1*). *Salmonella* usually causes a moderate gastrointestinal disorder, but it may result in more severe disease, such as bacteremia or meningitis, sometimes with fatal outcome (*2*,*3*).

For decades, reptiles have been recognized as a source of human salmonellosis. *Salmonella* species were first isolated from snakes, turtles, and lizards in the 1940s (*4*,*5*), and more recent studies have shown that at least 50%–90% of these animals are carriers of *Salmonella* (*6*–*8*). The bacteria are excreted intermittently in the feces but can also be isolated from the cloacae, skin, and throat of water-living reptiles.

Reptiles have become increasingly common as domestic pets. In Canada, pet turtle–associated salmonellosis was recognized as a serious health problem in the 1960s and 1970s, and the country banned imported turtles in 1975 (*9*).

Sweden has a long tradition of combating and controlling *Salmonella* in feed, animals, and humans, dating back to a large outbreak of salmonellosis in 1953 that affected >9,000 persons and caused 90 deaths (*10*,*11*). From 1970 to 1994, these control measures also included import restrictions on reptiles; anyone who wanted to import reptiles or turtles needed a certificate stating that the animals were free of *Salmonella*, and importing of turtles with shells <10 cm was not allowed.

In 1995, Sweden dropped its requirement for a *Salmonella* certificate and instead required an import permit issued by the Swedish Board of Agriculture. When Sweden became a member of the European Union (EU) in January 1995, a number of new rules were adopted. As a result of these changes, Sweden no longer required import permits for reptiles and turtles, and it also lifted the import ban on small turtles. The adaptation of import regulations for reptiles took effect on March 1, 1996.

Our study had 2 goals. First, we studied the impact of strict import regulations on the epidemiology of reptile-associated salmonellosis (RAS). Secondly, we assessed whether awareness campaigns can decrease the number of such cases.

## Methods

### Swedish Surveillance System for Salmonellosis

Salmonellosis is a reportable disease in Sweden. Case-patients, who need to have a *Salmonella*-positive stool or blood sample confirmed by a laboratory, are reported both by the physician who has seen the patient (clinical notification) and the laboratory that identified the bacterium (laboratory notification). Notification is submitted concurrently to the Swedish Institute for Infectious Disease Control, the county medical officer, and the municipal Environmental Health Board. Clinical notification includes relevant epidemiologic information, including suspected source of infection. If the county medical officer’s initial investigation indicates an environmental source of infection (such as food, water, or animals), the Environmental Health Board samples the suspected source.

### Reptile-associated Cases

We reviewed all reported domestic cases (patient reported to be infected in Sweden) of salmonellosis from 1990 to 2000 for association with reptiles. A patient was considered to have RAS if reference was made to direct or indirect contact with a reptile or turtle, and the notification did not indicate other sources of infection. All *Salmonella* strains were sent to the national Salmonella Reference Laboratory at the Swedish Institute for Infectious Disease Control, which performed serotyping according to the Kauffman and White scheme (*12*). In the case of *Salmonella enterica* serotype Typhimurium or *S.* Enteritidis, the laboratory also carried out phage typing according to the Colindale scheme. The Department of Epidemiology at the Swedish Institute for Infectious Disease Control compiled and analyzed all of these sources of information, together with reports on outbreaks from county medical officers.

All data used in this study were compiled as part of routine national surveillance of communicable diseases, as regulated in the Swedish Communicable Disease Act. The research ethics committee at Karolinska Institutet, Stockholm, Sweden, approved the study.

## Results

### Age and Sex of Patients

**Figure 1:**
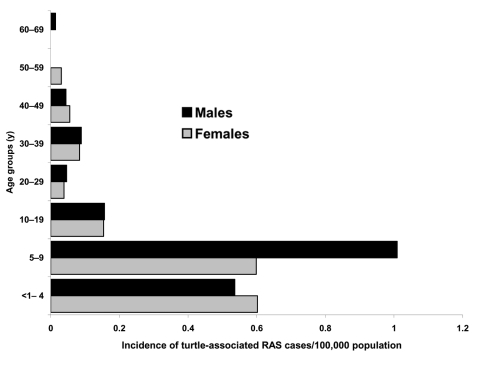
Age and sex distribution in the turtle-associated salmonellosis cases. RAS, reptile-associated salmonellosis.

**Figure 2:**
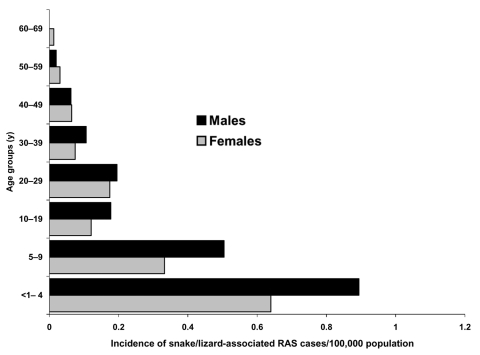
Age and sex distribution in the snake/lizard-associated salmonellosis cases. RAS, reptile-associated salmonellosis.

Clinicians and laboratories reported a total of 339 RAS cases in Sweden from 1990 to 2000. Patients infected by turtles (n = 153) were younger (median age 8 years, mean age 16 years) than patients infected by lizards and snakes (n = 175) (median age 17 years, mean age 18 years) (Figures 1 and 2). Eleven case-patients had contact with both turtles and lizards or snakes. Before 1996, RAS was more common in male patients (65% of cases). This difference disappeared after 1996, and from 1996 to 2000, approximately as many female (47%) as male (53%) patients were affected (nonsignificant difference).

### Serotypes

A total of 51 different serotypes were isolated from RAS-infected patients (Table 1). *S.* Enteritidis was the most frequent serotype, accounting for 24% of reported serotypes, followed by *S.* Typhimurium with 9%. Some serotypes were unique for patients with RAS.

**Table 1:** Reported serotypes of salmonellosis acquired in Sweden, 1990–2000

Serotype	No. of RAS* patients	No. of non-RAS patients	% of all RAS cases	% of all patients infected in Sweden	Type of reptile (could be >1 reptile/case)
*Salmonella enterica* Serotype Enteritidis	81	2,345	23.9	3.3	Turtle (46); tortoise (2); lizard (12); snake (28)
*S.* Typhimurium	30	2,056	8.9	1.4	Turtle (11); lizard (7); snake (16)
*S.* Subspecies III	30	8	8.9	78.9	Turtle (5); lizard (5); snake (25)
*S.* Subspecies I	19	41	5.6	31.7	Turtle (7); lizard (4); snake (8)
*S.* Newport	15	100	4.4	13.0	Turtle (3); lizard (4); snake (9)
*S.* Poona	15	19	4.4	44.1	Turtle (9); lizard (1); snake (5)
*S.* Saintpaul	12	53	3.5	18.5	Turtle (12)
*S.* Braenderup	12	30	3.5	28.6	Turtle (8); lizard(4)
*S.* Stanley	11	147	3.2	7.0	Turtle (11)
*S.* Muenchen	10	30	3.0	25.0	Turtle (4); lizard (1); snake (5)
*S.* Java	9	49	2.7	15.5	Turtle (5); snake (2); several reptiles (2)
*S.* Oranienburg	6	84	1.8	6.7	Turtle (2); lizard (3); snake (3)
*S.* Litchfield	6	12	1.8	33.3	Turtle (6)
*S.* Subspecies	5	177	1.5	2.7	Turtle (4); lizard (1)
*S.* Adelaide	5	33	1.5	13.2	Snake (5)
*S.* Chester	5	11	1.5	31.3	Turtle (2); snake (3)
*S.* Bovismorbificans	4	350	1.2	1.1	Turtle (3); snake (1)
*S.* Hadar	4	140	1.2	2.8	Turtle (3); snake (1)
*S.* Telelkebir	4	4	1.2	50.0	Lizard (2); snake (2)
*S.* Montevideo	3	55	0.9	5.2	Turtle (2); lizard (1)
*S.* Subspecies II	3	12	0.9	20.0	Turtle (1); lizard (1)
*S.* Subspecies IV	3	2	0.9	60.0	Lizard (3)
*S.* Infantis	2	324	0.6	0.6	Snake (2)
*S.* Bredeney	2	56	0.6	3.4	Turtle (2)
*S.* Heidelberg	2	44	0.6	4.3	Turtle (2)
*S.* Panama	2	35	0.6	5.4	Lizard (1); snake (1)
*S.* Abony	2	17	0.6	10.5	Turtle (2)
*S.* Ituri	2	5	0.6	28.6	Snake (2)
*S.* Napoli	2	1	0.6	66.7	Lizard (1)
*S.* Bonn	2	0	0.6	100.0	Turtle (1)
*S.* Nima	2	0	0.6	100.0	Snake (1)
*S.* Shubra	2	0	0.6	100.0	Turtle (1)
*S.* Agona	1	429	0.3	0.2	Snake (1)
*S.* Virchow	1	152	0.3	0.7	Turtle (1)
*S.* Thompson	1	54	0.3	1.8	Snake (1)
*S.* Blockley	1	51	0.3	1.9	Lizard (1)
*S.* Mikawasima	1	27	0.3	3.6	Turtle (1)
*S.* Muenster	1	7	0.3	12.5	Snake (1)
*S.* Oslo	1	3	0.3	25.0	Lizard and snake (1)
*S.* Reading	1	3	0.3	25.0	Snake (1)
*S.* Ibadan	1	2	0.3	33.3	Snake (1)
*S.* Monschaui	1	2	0.3	33.3	Snake (1)
*S.* Rubislaw	1	2	0.3	33.3	Snake (1)
*S.* Urbana	1	2	0.3	33.3	Turtle (1)
*S.* Farmsen	1	1	0.3	50.0	Snake (1)
*S.* Pomona	1	1	0.3	50.0	Turtle (1)
*S.* Abaetetuba	1	0	0.3	100.0	Turtle and lizard (1)
*S.* Lome	1	0	0.3	100.0	Snake (1)
*S.* Matadi	1	0	0.3	100.0	Turtle (1)
*S.* Spartel	1	0	0.3	100.0	Lizard (1)
*S.* Tamberma	1	0	0.3	100.0	Snake (1)
*S.* Veijle	1	0	0.3	100.0	Snake (1)
*S.* Windemere	1	0	0.3	100.0	Snake (1)
>1 serotype†	4	‡	1.2	–	Lizard (1); snake (3)
Not typed	2	‡	0.6	–	Snake (2)
Total no. of cases	339	6,974	100	100	–

*RAS, reptile-associated case of *Salmonella* infection, human. †1 case respectively with *S*. Adelaide + *S*. Montevideo, *S*. Agoueve + *S*. Chester + *S*. Infantis, *S*. Ajiobo + *S*. Muenchen, and *S*. Mesbit + *S*. Montevideo. ‡Not available.

### Cases before and after Adaptation of Importation Rules

Sweden has ≈9 million inhabitants; 4,500–5,200 report *Salmonella* infections each year. Less than 20% of reported cases (400–900 per year) are domestically acquired in Sweden.

**Figure 3:**
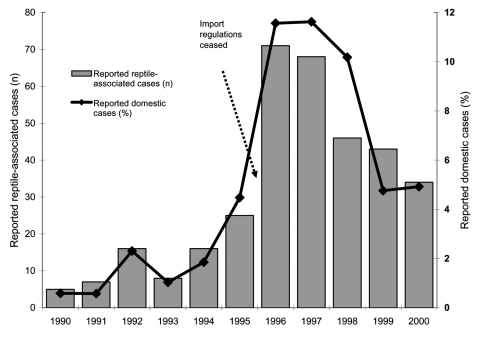
Reported cases of reptile-associated salmonellosis in Sweden, 1990–2000; total number of cases and proportion of domestic cases.

Table 2 and Figure 3 present the number of RAS cases in proportion to all domestic cases. From a very low proportion of RAS (1.2%, 5–16 cases) from 1990 to 1994 when import restrictions were in place, the proportion increased to 4.5% (25 cases) in 1995, as “*Salmonella* certificates” were no longer needed. The proportion of RAS increased even more (to 11.6%, 68–71 cases) in the 2 subsequent years, when all reptile import regulations had ceased.

**Table 2:** Reptile-associated salmonellosis and its proportion of all salmonellosis cases by study period, Sweden, 1990–2000*†

Study period	Incidence per 100,000 (no.) of all salmonellosis	Incidence per 100,000 (no.) of RAS cases	Proportion (%) RAS (95% CI)	p value
Period 1 (1990–1994)	12.74 (4,405)	0.15 (52)	1.2 (0.9–1.5)	<0.001
Period 2 (1995)	6.32 (558)	0.28 (25)	4.5 (2.9–6.5)	Reference
Period 3 (1996–1997)	6.78 (1,199)	0.79 (139)	11.6 (9.9–13.5)	<0.001
Period 4 (1998–2000)	7.70 (2,046)	0.46 (123)	6.0 (5.1–7.1)	NS

*Figures only include patients infected in Sweden. †RAS, reptile-associated salmonellosis; CI, confidence interval; NS, not significant.

In recognition of the growing problem with RAS, authorities alerted the public, mainly through the newspapers, starting in late 1997. In the 2 following years (1998–2000) the proportion of RAS cases decreased to 6.0% (43 and 34 cases), but it did not reach the low levels seen before 1995.

Of the 77 RAS cases diagnosed before the adaptation with EU importation rules in 1996, 26 (34%) persons had contracted the infection from turtles and 51 (66%) from snakes and lizards. After adaptation, the proportion of cases from the 2 sources was more even; of 262 diagnosed cases, 127 (48%) were due to contacts with turtles and 124 (47%) were due to contacts with snakes and lizards. Eleven (4%) of the case-patients after 1995 had a history of contact with several kinds of reptiles. Since no sampling of the animals was performed, we do not know which kind of reptile caused these infections.

## Discussion

First, this report shows that an import restriction requiring certificates that animals are not carrying *Salmonella* is an effective public health measure for protecting the general population from RAS. Sweden noted a dramatic increase in the number of reported cases of RAS in 1996. No changes in the reporting system were made that could explain the increase in reported cases, making it likely that synchronization of import rules of animals and reptiles within the EU in March 1996 caused the sudden increase in the number of RAS cases in Sweden that year.

Secondly, our report indicates that actively informing the public about RAS can cause a decrease in cases. Immediately after the harmonization of rules, the availability of reptiles in pet stores increased, and many parents gave their children a pet turtle, lizard, or snake. The exact number of animals sold is unknown. No information about the risk of contracting salmonellosis was given to the families who bought the animals. In the United States, the Association of Reptilian and Amphibian Veterinarians (ARAV) produced a client education handout with basic facts on how to avoid transmission of *Salmonella* bacteria from reptiles to humans (*13*). The Centers for Disease Control and Prevention recommends that children under the age of 5 should avoid contact with reptiles and that households with children <1 year should not own reptiles.

Information about the risk of contracting salmonellosis from pet reptiles was communicated to the public from late 1997 and onwards, and the information given to the public in Sweden has closely followed ARAV guidelines. After the information activities began, the number of reported RAS cases decreased significantly in subsequent years. The decrease might have been even greater since it is likely that more patients with diarrhea would have informed their doctors of reptile contact once they knew of the association between reptiles and salmonellosis.

Third, our study demonstrates that RAS poses a threat to human health that cannot be ignored. The true number of such cases is hard to estimate. We have little hard data on the proportion of all *Salmonella* cases in the community being diagnosed, although according to conservative estimates, ≈90% of cases remain undiagnosed (*11*). The Swedish system, with its dual notifications from clinicians and laboratories, captures >99% of cases actually being diagnosed, and >95% of all diagnosed cases are clinically reported (A. Jansson, unpub. data). We assume that at least 50% of RAS patients have been reported as such, once a *Salmonella* infection has been diagnosed, for the following reasons: 1). only a few clinical notifications lack information on likely route of transmission, 2) domestically acquired salmonellosis is comparatively rare, and 3) most physicians are aware of this route of transmission. We estimated the true annual number of RAS in Sweden after 1996 to be ≈1,000 cases per year, or >10 cases per 100,000 inhabitants. The same rate applied in the United States would correspond to 30,000 to 40,000 cases per year. This number is substantially less than the 93,000 cases per year estimated by the Centers for Disease Control and Prevention (*14*), which hypothesized that 7% of all U.S. cases of salmonellosis were RAS.

On the basis of these assumptions, we estimated the true annual number of RAS in Sweden after 1996 would be ≈1,000 cases per year, or >10 cases per 100,000 inhabitants. This estimate should correspond to 30,000–40,000 cases in the United States, which is less than the estimated 93,000 RAS cases per year in the United States, accounting for 7% of all cases of salmonellosis (*14*).

The incidence of RAS in a country is naturally dependent on the magnitude of reptile imports. We therefore tried to obtain some estimates of the numbers of imported reptiles in the years under study. This attempt was unsuccessful because the responsible national authority (the Swedish Board of Agriculture) has no figures on the number of imported animals, only records on the importer. Furthermore, according to direct private import rules, anyone can legally bring <3 animals into the country without any registration. Although we have no indication that the numbers of imported reptiles decreased in the later years (rather, we think the opposite is true), we cannot rule out that some of the decrease in RAS cases was due to decreased imports.

Fourth, we obtained information on the epidemiology of RAS that is likely to hold true for other Western countries as well. Children are the most affected age group, with an incidence of 1.3/100,000 inhabitants. Those patients who had acquired salmonellosis from a turtle were younger than patients who acquired it from a snake or lizard, a reflection of the age groups for which turtles are bought as pets. Young boys 5 to 9 years of age who had contracted the disease from a turtle were shown to be at particular risk in this study.

In the United States during the 1970s, small pet turtles were a major source of RAS infections and accounted for 14% of all cases of salmonellosis in children <10 years of age. This fact was the reason for the ban on commercial distribution of pet turtles with a carapace length <4 inches. This ban has prevented an estimated 100,000 RAS cases among children in the United States yearly (*14*,*15*). Nevertheless, the incidence of RAS in the United States has been increasing because of an increase in pet reptile owners; experts estimate that the number of pet reptiles owners doubled from 1991 to 2001 (*3*).

In most cases included in this study, patients experienced gastrointestinal infection with symptoms severe enough to seek medical care; some even required hospitalization. Many of the serotypes isolated from the Swedish RAS patients were serotypes also commonly found in foodborne *Salmonella* infection. *S.* serotype Enteritidis and *S.* Typhimurium, the 2 most common serotypes found in humans in Europe and the United States (*16*,*17*), accounted for 33% of all RAS cases, while *S*. Subspecies III, known to be reptile-associated, caused 9% of cases. Some serotypes such as *S.* Abaetetuba, *S.* Bonn, *S.* Lome, *S.* Matadi, *S.* Nima, *S.* Shubra, *S.* Spartel, *S.* Tamberma, *S.* Veijle, and *S.* Windemere were only found in RAS cases and could therefore be assumed to be more reptile-associated than other serotypes. Previously known reptile-associated serotypes, including *S.* Poona, *S.* Stanley, *S.* Pomona, and *S.* Java, were also isolated from Swedish RAS patients (*15*). Some serotypes may be more species-specific; 24 of the 51 isolated serotypes were isolated from only 1 kind of reptile (turtle, snake, or lizard). In most of these cases, the numbers were too low to allow for any firm conclusions. However, *S.* Litchfield, *S.* Saintpaul, and *S.* Stanley seem to be associated with turtles and *S.* Adelaide with snakes. The wide variety of *Salmonella* serotypes in persons with RAS demonstrates that reptiles are well adapted to *Salmonella* and could harbor and transmit many different serotypes, which confirms that a risk is always involved in handling these kinds of animals.

Having the same close contact with pet reptiles and turtles as with cats and dogs, or even just keeping them in the private home, increases the risk for transmission of *Salmonella* bacteria from animals to humans. Pet stores, veterinarians, and other appropriate sources should provide better information on how to avoid these risks, such as the ARAV guidelines, to all prospective buyers of turtles and other reptiles.
